# More people, more cats, more parasites: Human population density and temperature variation predict prevalence of *Toxoplasma gondii* oocyst shedding in free-ranging domestic and wild felids

**DOI:** 10.1371/journal.pone.0286808

**Published:** 2023-06-21

**Authors:** Sophie Zhu, Elizabeth VanWormer, Karen Shapiro

**Affiliations:** 1 Department of Pathology, Microbiology, and Immunology, University of California, Davis, Davis, CA, United States of America; 2 School of Veterinary Medicine and Biomedical Sciences, University of Nebraska-Lincoln, Lincoln, Nebraska, United States of America; 3 School of Natural Resources, University of Nebraska-Lincoln, Lincoln, Nebraska, United States of America; Universidad Santo Tomas, CHILE

## Abstract

*Toxoplasma gondii* is a ubiquitous zoonotic parasite that can infect warm-blooded vertebrates, including humans. Felids, the definitive hosts, drive *T*. *gondii* infections by shedding the environmentally resistant stage of the parasite (oocysts) in their feces. Few studies characterize the role of climate and anthropogenic factors in oocyst shedding among free-ranging felids, which are responsible for the majority of environmental contamination. We determined how climate and anthropogenic factors influence oocyst shedding in free-ranging domestic cats and wild felids using generalized linear mixed models. *T*. *gondii* oocyst shedding data from 47 studies were systematically reviewed and compiled for domestic cats and six wild felid species, encompassing 256 positives out of 9,635 total fecal samples. Shedding prevalence in domestic cats and wild felids was positively associated with human population density at the sampling location. Larger mean diurnal temperature range was associated with more shedding among domestic cats and warmer temperature in the driest quarter was associated with lower oocyst shedding in wild felids. Increasing human population density and temperature fluctuation can exacerbate environmental contamination with the protozoan parasite *T*. *gondii*. Management of free-ranging domestic cats could lower the burden of environmental oocysts due to their large population sizes and affinity with human settlements.

## Introduction

Climate change has not only led to devastating changes in ecosystem function and provided services but has also facilitated the emergence and/or expansion of many human and animal pathogens worldwide [1]. The reasons for disease emergence and expansion are complex and multifaceted but can include expanded pathogen or vector range, altered species interactions, and reduced host fitness [2, 3]. Understanding the climatic and ecological drivers of pathogen spread is vital for the prevention and mitigation of diseases in both animal and human hosts. *Toxoplasma gondii* is a generalist zoonotic pathogen that can infect any warm-blooded vertebrate, including humans [4]; however, it can only sexually reproduce in its definitive hosts–domestic cats and wild felids [5]. The global distribution and large population of domestic cats (˜600 million) [6], coupled with numerous parasite transmission pathways, contributes to *T*. *gondii*’s ubiquitous nature and infection in hosts from remote oceanic islands to the Arctic Circle [7, 8].

Toxoplasmosis can cause mild-to-severe disease in humans, domestic animals, and wildlife. Human infections are generally less common in most high-income nations, but toxoplasmosis is still a large contributor to disease burden in many regions. Disease burden is particularly high in South America, which has a high diversity of *T*. *gondii* genotypes, high levels of environmental contamination, and large populations of free-ranging domestic cats and wild felids [9]. Toxoplasmosis can also cause abortion in sheep and goats, which can be a financial burden for farmers [10]. In addition, marine wildlife can be vulnerable to *T*. *gondii* infections; oocysts accumulate in soil and flush into marine environments after rainfall, whereby they can contaminate prey sources consumed by susceptible hosts [11]. Domestic and wild felid species play a critical role in the ecology and epidemiology of *T*. *gondii* because they are the only source of oocysts, the parasite life stage that drives overall *T*. *gondii* transmission [12].

Cats typically shed oocysts for 1–3 weeks after primary infection, and research has been conducted in controlled, experimental settings to investigate risk factors for shedding in domestic cats, which include route of infection, age, and immunosuppression [13–15]. Previous reports have suggested that 1% of all domestic cats are estimated to be shedding at any given point [16], though a recent meta-analysis reported a global pooled oocyst shedding prevalence of 2.6% (95% CI 1.9–3.3) for domestic cats [17]. The commonly cited 1% oocyst shedding prevalence likely underestimates shedding as it does not take into account the potential for re-shedding oocysts, and differences among cats, including diet and time spent outdoors. Outdoor pet cats, managed free-ranging cat colonies, truly feral/stray cats, and wild felid species that rely on partial or exclusively hunted prey have a higher risk of infection from consuming infected intermediate hosts and contact with oocyst-contaminated matrices. These felid populations are more frequently exposed to diverse genotypes of *T*. *gondii*, which may enhance the potential for initial and repeat oocyst shedding [18]. Less attention has been directed at investigating risk factors for shedding prevalence in free-ranging wild and domestic felids compared to owned domestic cats, despite the importance of the former groups in contributing to environmental contamination and toxoplasmosis transmission.

Temperature, precipitation, and humidity can impact oocyst sporulation and survival, oocyst transport/mobilization (via rainfall patterns), and felid host distribution, reproduction, and population size [19] (S1 Fig in S1 File). Climate can also affect the life cycle and population dynamics of common prey species, which can provide favorable conditions for cat reproduction and increased numbers of young, naive definitive hosts susceptible to *T*. *gondii* infection [20].

Using published data on *T*. *gondii* oocyst shedding in free-ranging domestic and wild felids, we investigated whether climate and anthropogenic risk factors are predictive of shedding prevalence. Other studies have evaluated risk factors in a specific location but, to our knowledge, this study is the first to use a global dataset and consider both climate and anthropogenic factors. To investigate the impacts of temperature, precipitation, and anthropogenic activity on *T*. *gondii* oocyst shedding in free-ranging felids, we hypothesized that shedding will increase with warmer temperatures, higher precipitation, and in areas with higher human population density. Our study builds upon previous research by investigating risk factors for shedding in both free-ranging wild felids and domestic cats, the latter of which play a key role in environmental contamination with oocysts in many areas and can be more readily targeted in management and policy efforts.

## Methods

### Literature search

Our analyses focused on confirmed *T*. *gondii* oocyst shedding in domestic and wild felids. A systematic review of the literature was performed in March 2020 using PubMed and Web of Science, with a subsequent sweep in July 2021 (n = 2176). Twelve sets of search terms were used, always including ‘*Toxoplasma*’ (See S1 Table of Zhu et al. 2021 [21]). All studies through July 2021 were included, and no studies were excluded based on language of publication or location. Google Translate was used to translate and extract metadata as necessary. We obtained additional studies from the reference lists of reviews identified in this sweep along with a recent meta-analysis (n = 51) [17], for a total of 2227 studies. Duplicates (n = 1111), reports not able to be retrieved (n = 2), and reports that did not focus on *T*. *gondii* or *T*. *gondii*-like oocyst shedding in felids were excluded (n = 1003). Studies were restricted to oocyst shedding in free-ranging wild felids and unowned, free-ranging domestic cats, which include stray, semi-managed feral cats (i.e. unowned cats that are fed by humans but do not live indoors), and unmanaged feral cats that subsist on wild prey without human support. One study included cats euthanized at a humane shelter in Ohio [22]. According to a national database of 1,233 animal shelters in the US, the majority of live intakes for cats are strays, or unowned or free roaming animals [23]. For analysis, we assumed that the majority of unowned cats from the Ohio shelter would be stray or free-ranging, similar to values reported by the national database, though we acknowledge that we could not verify the exact origin of all animals. When fecal samples are screened using only fecal flotation and microscopy, *T*. *gondii* cannot be reliably distinguished from related apicomplexans, namely *Hammondia hammondi*. Confirmation of oocysts as *T*. *gondii* via PCR with sequencing or mouse bioassay is necessary to confirm parasite identity. We only included studies that verified oocyst presence with microscopy coupled with genetic parasite identification using PCR and/or mouse bioassay. After removing publications based on exclusion criteria for oocyst confirmation (n = 64), 47 studies were included in the final analysis (S2 Fig in S1 File). For studies that met inclusion criteria, we extracted metadata including the year of publication, type of felid sampled (domestic/wild), country of study, continent, diagnostic method, number of positive fecal samples, total fecal samples tested, and approximate latitude and longitude of the sample site as reported by the authors for each study. We repeated this process in studies with multiple species of sampled felids. All data sources are provided in S1 Appendix and a table with summarized metadata and all variables considered in analysis for each study was uploaded to DataDryad [24].

Our primary variables of interest were annual mean temperature, annual precipitation, and human population density. Additionally, we considered other climate variables, such as maximum temperature, mean diurnal temperature range and precipitation seasonality, as well as human activity variables such as habitat type and species richness for inclusion in subsequent models (Table 1). For each study, we used the R package ‘raster’ [25] with a World Geodetic System 1984 projection and a 2.5 km buffer to obtain location-specific data. Climate data (temperature and precipitation variables) was extracted at a 5 km resolution from the WorldClim 2.0 (1970–2000) dataset [26], while human population density and human footprint data at a 5 km resolution was extracted from the NASA Center for International Earth Science Information Network (CIESIN) [27]. Human population density was paired to each study by the closest time period (2000, 2005, 2010, 2015 and 2020). Habitat type was extracted at a resolution of 1 km, and species richness at 110 m from the International Union for Conservation of Nature (IUCN) [28] (Table 1).

**Table 1:** Description, rationale, and data source for variables assessed in univariable analysis and multivariable model building as predictors of *Toxoplasma gondii* oocyst shedding in free-ranging felids.

Variable Type	Rationale	Available data	Data source(s)
Temperature	Differences in temperature may alter oocyst survival [28]	Annual mean temperature **Mean diurnal temperature range (D)** Isothermality Temperature seasonality Max temperature of warmest month Min temperature of coldest month Temperature annual range Mean temperature of wettest quarter Mean temperature of coldest quarter **Mean temperature of driest quarter (W)** Mean temperature of wettest quarter	WorldClim [26]
Precipitation	Precipitation can affect oocyst survival and transport [29–31]	Annual precipitation Precipitation of wettest month Precipitation of driest month Precipitation seasonality Precipitation of wettest quarter Precipitation of driest quarter Precipitation of warmest quarter Precipitation of coldest quarter Vapor pressure	WorldClim [26]
Human activity	Anthropogenic activities and human presence can alter landscapes in ways that lead to changes in domestic and wild felid population size and density, and diversity of intermediate hosts.	IUCN habitat type Species richness **Human population density (D/W)** Human footprint	IUCN [28], NASA CIESIN Gridded Population of the World (GPW) v4 [27]

Variables included in final multivariable models for domestic (D) and wild (W) felids are **bolded** for emphasis.

### Statistical analysis

We tested our hypotheses using generalized linear mixed models implemented in the R package ‘glmmTMB’ [32]. We chose to restrict our analysis to studies of confirmed *T*. *gondii* oocyst shedding (microscopy coupled with parasite confirmation via PCR and/or mouse bioassay) because climate and seasonality can have dissimilar relationships with shedding patterns of *T*. *gondii* and *T*. *gondii-*like oocysts (such as *H*. *hammondii*) [33]. *T*. *gondii* oocyst shedding prevalence was modeled as a proportion between 0 and 1 with a beta distribution. Exploratory analyses showed that the data were zero-inflated, resulting in the use of a zero-inflated beta distribution for further analyses. To account for other sources of variance such as spatial autocorrelation, we incorporated the study as a random effect in all multivariable models.

Model building and comparison were performed for domestic and wild felid data separately as the distribution of associated risk factors was different between the two groups based on descriptive statistics, possibly resulting from a combination of sampling bias and biological differences in size, habitat, and dietary habits. The distribution of habitat type, species richness, and human population density for domestic and wild felids are summarized in S3 Fig in S1 File. All variables were standardized and scaled prior to analyses. Variables were evaluated one at a time and considered for inclusion in multivariable models using a significance threshold of p<0.10. As there were multiple highly correlated variables used to measure temperature and precipitation, final variables were selected by category (temperature/ precipitation/anthropogenic activity) based on strength of significance using a threshold of p<0.01 (S1 Table in S1 File). We calculated Pearson correlation coefficients between final variables; no variables tested had a coefficient above 0.4 (S2 and S3 Tables in S1 File). There were several temperature variables (e.g., temperature seasonality, temperature annual range, mean temperature in dry quarter) with p<0.01 for wild felids, and two temperature variables with p<0.01 for domestic cats, so we tested these factors in separate multivariable models and compared model AIC to determine final model composition. We did not evaluate interactions between candidate variables and our outcome because we expected linear relationships. The best model for each felid group (wild or domestic) was chosen based on AIC.

## Results

The final dataset of only confirmed *T*. *gondii* oocysts included 51 groups of felids sampled from 47 studies (256 positives out of 9,635 fecal samples) (S1 Appendix) [24]. Sample size and oocyst shedding prevalence varied by felid type, with 37 out of 463 (8%) fecal samples testing positive from 12 groups of wild felid species sampled from 10 studies, and 219 out of 9,172 (2.4%) fecal samples testing positive from 39 groups of domestic cats sampled across 37 studies. The six wild felid species represented in our study include bobcat (*Lynx rufus*), cougar (*Felis concolor*), Geoffrey’s cat (*Oncifelis geoffroyi*), Pampas cat (*Felis colocolo*), Iriomote cat (*Prionailurus iriomotensis*), and Pallas’s cat (*Otocolobus manul*).

**Fig 1:**
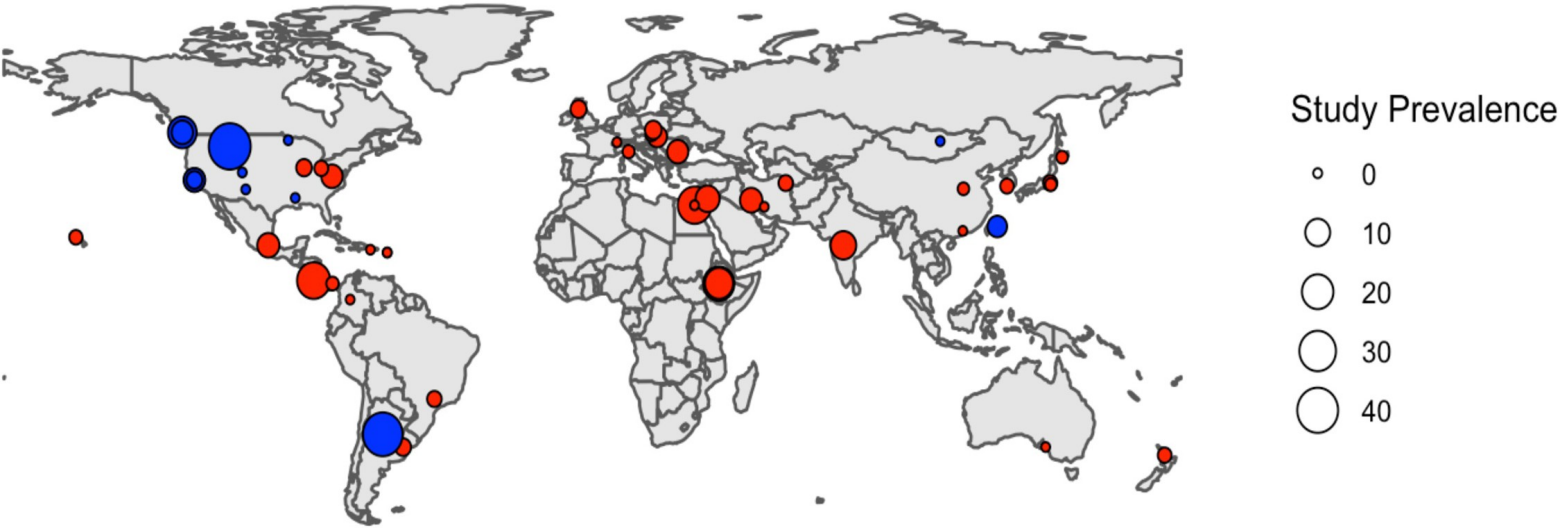
Map of confirmed *T*. *gondii* oocyst shedding prevalence in free-ranging domestic cats (red) and wild felid species (blue) in studies conducted from 1973–2021. Modified from Zhu et al. 2021 [21] and included for illustrative purposes. Map created using ‘rnaturalearth’ package [34].

Domestic cat studies were primarily conducted in North America (n = 11), Asia (n = 11), and Europe (n = 7), with fewer studies conducted in Africa (n = 4), South America (n = 3), and Oceania (n = 2). The majority of shedding studies with confirmed *T*. *gondii* in feces from wild felids were conducted in North America (n = 7), with two studies conducted in Asia and one study in South America (Fig 1). The majority of domestic cat studies (n = 29/38, 76%) were conducted in human-modified/urban environments. Non-urban habitats included forest-temperate (n = 5), forest-subtropical/tropical moist lowland (n = 1), forest-subtropical/tropical moist montane (n = 1), savanna-dry (n = 1), shrubland (n = 1), shrubland-Mediterranean (n = 2), and grassland—subtropical/tropical dry (n = 1). Most wild felid studies were conducted in non-urban habitats, namely forest-temperate (n = 5), shrubland-temperate (n = 1), shrubland-subtropical/tropical dry (n = 1), shrubland-Mediterranean (n = 2), and grassland-temperate (n = 1). Two groups of wild felids were sampled in agricultural environments.

Based on univariable analysis, four variables were retained in multivariable model building for free-ranging domestic cats (mean diurnal temperature range, isothermality, precipitation seasonality, human population density) and four variables for wild felids (temperature seasonality, temperature annual range, mean temperature in dry quarter, human population density). A total of twelve linear models were considered to explain confirmed *T*. *gondii* oocyst shedding in domestic cats (S3 Table in S1 File). The top model, based on AIC, showed a significant, positive effect of human population density (OR 1.38, p = 0.0014) and mean diurnal temperature range (OR 1.63, p = 0.0007) on *T*. *gondii* oocyst shedding prevalence in domestic felids. Eight models were considered to explain oocyst shedding in wild felids (S4 Table in S1 File). Between two wild felid models with the same AIC value, we chose the model with population density and mean temperature in the driest quarter because it had no significant deviations for actual vs. predicted residuals. The top model showed a positive association between human population density (OR 340, p = 1.54 e-11) and a negative association between mean temperature in the driest quarter (OR 0.26, p<2e-16) and oocyst shedding in wild felids (Table 2).

**Table 2:** Final multivariable model structure and effect size (*β* and OR) for evaluation of association between climate (temperature, precipitation) and anthropogenic risk factors of domestic cat and wild felid oocyst shedding.

Model type	Model variable(s)	Beta	OR (95% CI)	p-value
Domestic cat^a^	Human population density	0.32	1.38 (1.13, 1.68)	0.0014
	Mean diurnal temperature range	0.49	1.63 (1.22, 2.17)	0.0007
Wild felid^a^	Human population density	5.34	340 (62, 1856)	1.54 e-11
	Mean temperature in dry quarter	-1.34	0.26 (0.19, 0.36)	2 e-16

^a^Study was also included as a random effect in both models for domestic and wild felids to account for other unaccounted sources of variance.

## Discussion

We found a significant positive association between human population density and *T*. *gondii* oocyst shedding prevalence in a global dataset of free-ranging domestic cats and six wild felid species (S1 Appendix) [24]. Other variables of interest, namely total annual precipitation and mean annual temperature, were not associated with *T*. *gondii* oocyst shedding prevalence. Although lower prevalence of oocyst shedding has been reported in domestic cats versus wild felids [17, 21], their large population sizes and association with human populations mean that they are an important source of *T*. *gondii* oocysts for wildlife and people. As global estimates of domestic cat population density do not exist, human population density offers a proxy within our models as higher human density and activity can result in the release of unwanted pets, more outdoor pet cats, pet cats escaping, and proffered feeding to feral cat colonies. Additionally, increasing human activity can lead to landscape changes that may facilitate increased environmental oocyst contamination [35]. Taken together, these factors can alter the epidemiology and transmission of *T*. *gondii* by facilitating the increased abundance of free-ranging domestic cats that contribute to oocyst contamination in the environment.

Our main finding that human density is significantly associated with oocyst shedding prevalence in domestic and wild felids is consistent with a recent study that found associations between human population density and *T*. *gondii* seroprevalence in wild mammals [36]. Association with human settlements introduces a multitude of behaviors that benefit feral cats through the provision of additional food, deterrents to native predators, and protection against the elements, which in turn could help to maintain and even increase colony and overall cat population size. Human populations are concentrated in urban areas, which have increased availability of food (trash, intentional food sources like bird feeders) that can attract urban wildlife like rats and birds [37]. Higher prey populations can support larger populations of free-ranging cats, so even low seroprevalence rates in prey (<1%) can result in widespread cat infection with *T*. *gondii* due to the sheer number of prey consumed and high likelihood of consuming infected prey over time [38]. Though access to additional anthropogenic food could reduce prey consumption by urban cats, direct and indirect contact (such as sharing fecal latrines) among individuals can facilitate pathogen transmission in dense urban cat populations [39]. Wild felids that live near urban-rural interfaces have increased opportunities to share a food web with domestic felids and can be exposed to infected commensal intermediate wildlife hosts like rodents as well as *T*. *gondii* strains commonly associated with domestic cats in addition to wild strains. Oocyst spread is also facilitated in human-dominated landscapes. Modified surfaces like roads allow surface water runoff to transport oocysts easily from land into sources of water shared by humans and animals [30], whereas natural cover and vegetation can reduce oocyst delivery by filtering pathogens in runoff [40].

Oocyst load in the environment is not sufficient by itself to estimate infection risk because host and environmental factors can interact to influence location-specific risk. Our measured outcome was the prevalence of *T*. *gondii* oocyst shedding (generally only occurring for 1–2 weeks), not the prevalence of *T*. *gondii* infection, which is more easily captured due to presence of antibodies due to lifelong infection. Prior studies of *T*. *gondii* in wild mammals found a positive association between mean annual temperature and seroprevalence, however, infection in intermediate hosts and shedding by definitive hosts are related but separate phenomena that may be impacted differentially by climate, especially at different time scales [36]. Climate data sets used in this study spanned a 30 year period and were not necessarily designed to assess seasonality [26]. While our analysis did not reveal clear relationships between oocyst shedding and mean annual temperature or precipitation, warmer temperatures and higher humidity are linked to higher *T*. *gondii* infection prevalence in humans and livestock [41, 42], and higher cat seroprevalence has been linked to greater local rainfall [43]. Mechanistically we still do not understand how variables such as mean diurnal temperature range directly influence oocyst shedding, however we recommend that the impacts of climate on *T*. *gondii* oocyst shedding should be assessed in longitudinal studies at the local scale in order to gain a clearer picture of this relationship.

One limitation of our analysis is that many primary literature sources did not perform serological testing to discriminate between animals with previous evidence of exposure (e.g. antibodies produced against *T*. *gondii*). In two of the original studies [44, 45], most of the domestic and wild felids were seropositive, indicating that they had most likely already shed oocysts at least once. Though seropositive cats can shed oocysts [21, 46], studies with seropositive animals may be less likely to capture oocyst shedding than studies sampling naïve animals. Two other limitations of this study and similar reports on *T*. *gondii* oocyst shedding are geographic and species-based sampling bias, and inconsistency of detection methods used to determine oocyst shedding. Due to the small sample size and large confidence interval for effect size in wild felids (62–1856), we caution that our wild felid results may be incredibly sensitive to sampling bias and may need further investigation to determine the true association with human population density. Despite the diversity of *T*. *gondii* genotypes present in South America, oocyst shedding in this region remains critically understudied. Studies are also sparse in Africa and Asia, even though both regions have multiple wild felid species and high projected human population growth [47]. Africa is consistently reported to have the highest confirmed and non-confirmed oocyst shedding prevalence compared to other continents [17, 21] (Fig 1). As human population density continues to increase in these regions, it is necessary to monitor environmental *T*. *gondii* oocyst contamination as it is relevant to human and wildlife health [48]. Future studies should also consider socioeconomic status (SES) to understand how people can be disproportionately affected by urbanization and disease transmission. Wealthy neighborhoods often have sufficient resources to manage populations of unwanted rodents through culling and proper sanitation measures, which indirectly helps to deter stray and feral cats. Water infrastructure in resource-limited countries can also be heterogeneous. In Brazil, richer individuals may have access to treated and/or bottled water while those living in favelas (urban slums) do not [49]. Similar observations have been reported for *Toxocara* infections in the boroughs of New York City [50], increased *T*. *gondii* seropositivity among lower SES groups in the US [51], and higher rates of maternal exposure to *T*. *gondii* and congenital toxoplasmosis in Brazil among lower SES groups [52]. The challenge of variable fecal testing methods and sampling bias exacerbates inequality, as there are more microscopy-based studies in low and middle-income countries (LMICs) compared with North America and Europe. From an equity lens, microscopy-based studies are biased toward LMIC laboratories that have less funding and resources compared to laboratories in high-income nations that are well-resourced, well-financed, and have higher training ability that can perform more molecular and bioassay studies. We thus have a clearer picture of *T*. *gondii* transmission in regions that are relatively low risk for *T*. *gondii* exposure and an ambiguous picture where high-quality data may actually be more valuable to protect human public health and vulnerable animal populations.

Identification of predictive and/or protective factors for oocyst shedding in free-ranging wild and domestic felids is highly relevant for stakeholders and policymakers to make informed decisions for human and animal health. Our results reinforce the role that free-ranging domestic cats have in contributing to biological contamination of *T*. *gondii* oocysts, and therefore the need to target management (cat removal) and/or more holistic landscape-based interventions (wetland restoration, vegetation buffers) that can reduce transport of oocysts [40, 53]. Overall, we recommend that more studies be conducted in locations where *T*. *gondii* is highly endemic but understudied such as South America and Africa, the utilization of molecular methods paired with microscopy to verify parasite identity and characterize oocyst genotypes, and more sampling of wild felid species. Most regions are under sampled for wild felids, despite the fact that a higher proportion of these animals are thought to be shedding at any given point in time. We do not attempt to prove a causal relationship between human population density and oocyst shedding prevalence, however, the association between human density and oocyst shedding suggests that focusing on free-ranging feral cats as the source of environmental oocyst contamination is an important disease management strategy. Feral cat management can serve to reduce the risk of toxoplasmosis in humans, livestock, and wildlife, and will also have added benefits for reducing predation of native wildlife species [54]. We emphasize the need to address major data gaps and potential strategies for monitoring *T*. *gondii* oocyst shedding prevalence in free-ranging domestic cat and wild felid populations. Human-caused climate change and urbanization may create more ideal environmental conditions for a generalist pathogen and its disease ecology, which can be relevant for other infectious diseases that are influenced by human population growth and increased urbanization.

## Supporting information

S1 File Please see attached files for S1-S3 Figs and S1-S4 Tables. (DOCX)

S1 Appendix Reference list of all studies included in multivariable analysis of *T*. *gondii* oocyst shedding in free-ranging domestic and wild felids. (DOCX)
