# Supplementary material for: More people, more cats, more parasites: Human population density and temperature variation predict prevalence of Toxoplasma gondii oocyst shedding in free-ranging domestic and wild felids
Source: PLoS One. 2023 Jun 21;18(6):e0286808. doi: 10.1371/journal.pone.0286808 (PMC10284397; doi:10.1371/journal.pone.0286808)
Supplement: S1 File — (DOCX) [file pone.0286808.s001.docx]

**S1 Fig. Mechanistic framework of pathways in which climate and anthropogenic factors can influence *Toxoplasma gondii* oocyst shedding in free-ranging domestic cats and wild felids.**

**
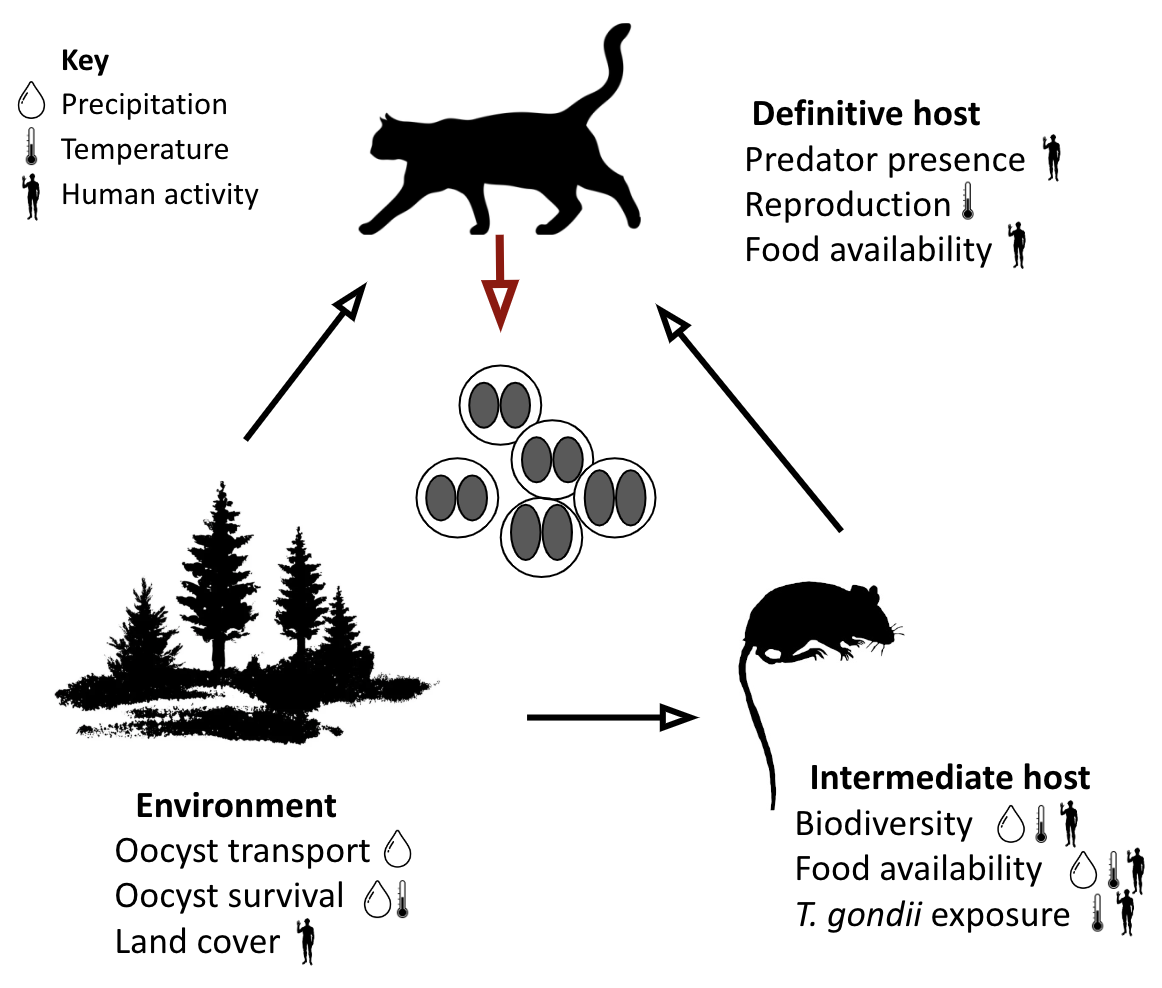
**

Each parameter that can affect *T. gondii* transmission (and thus felid oocyst shedding) can be influenced by precipitation, temperature and/or human activity, as noted by the symbols listed in the key.

**S2 Fig. Preferred Reporting Items for Systematic Reviews and Meta-Analyses (PRISMA) 2020 flow diagram.**

**
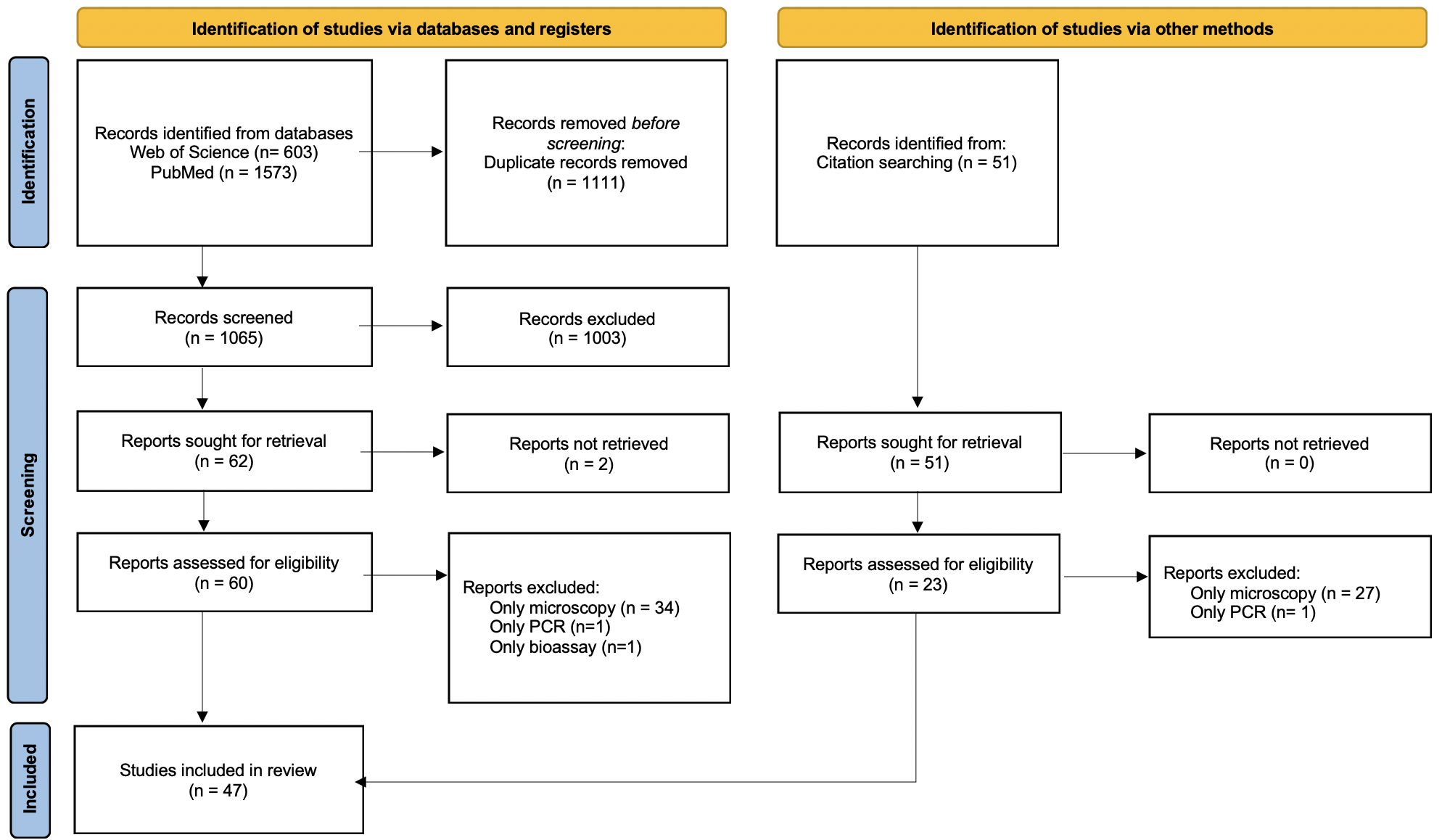
**

The following PRISMA flow diagram describes the identification, screening, and inclusion process for the 47 studies analyzed in this manuscript. *From*: Page MJ, McKenzie JE, Bossuyt PM, Boutron I, Hoffmann TC, Mulrow CD, et al. The PRISMA 2020 statement: an updated guideline for reporting systematic reviews. BMJ 2021;372:n71. doi: 10.1136/bmj.n71. For more information, visit: <http://www.prisma-statement.org/>

**S3 Fig. Distribution of scaled and standardized a) annual mean temperature (℃), b) annual precipitation (mm), c) IUCN habitat type, d) species richness, and e) human population density (people/km^2^) in studies reporting the prevalence of oocyst shedding by free-ranging domestic cats (red) and wild felids (blue).**

| a) Annual mean temperature (℃) | b) Annual precipitation (mm) |
| --- | --- |
| 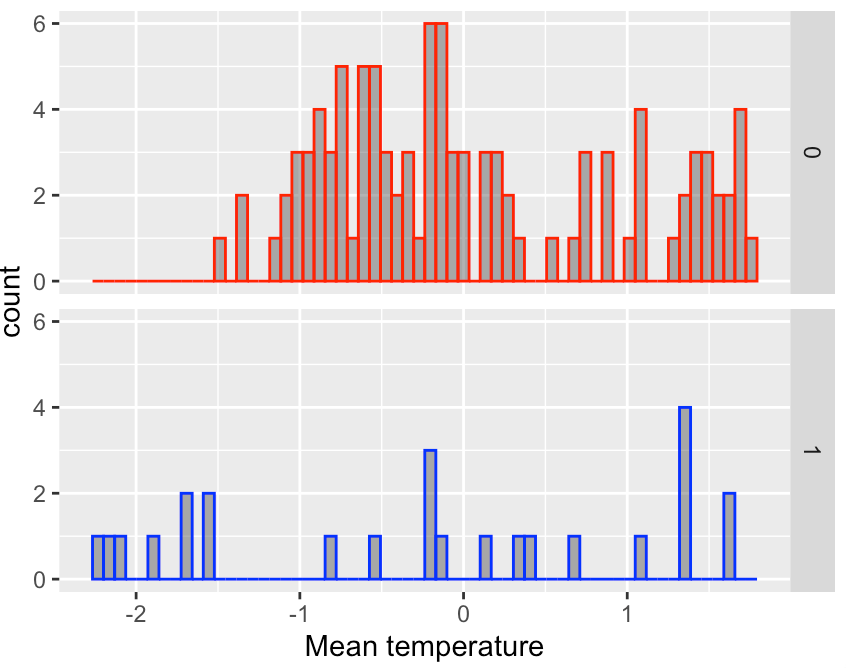 | 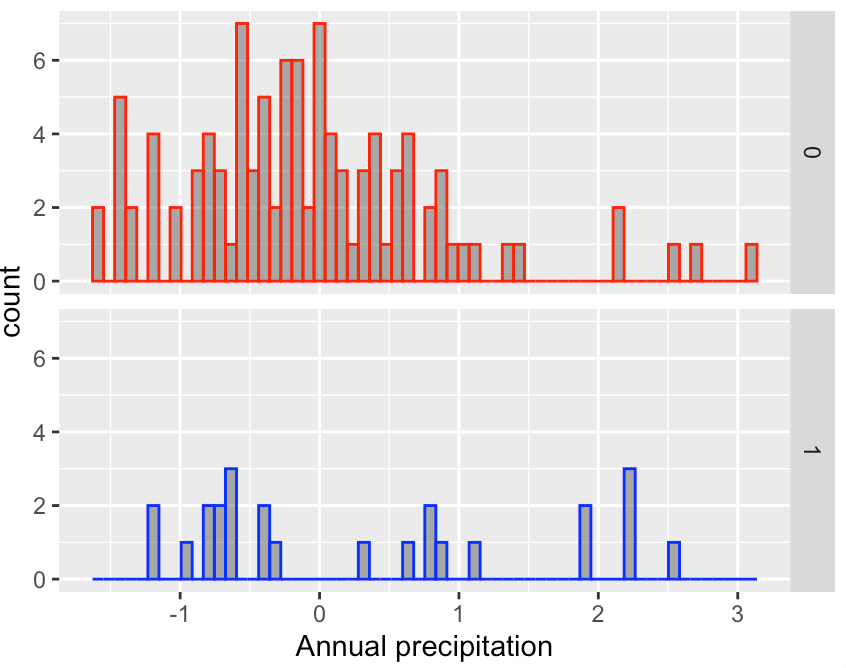 |
| c) IUCN habitat type | d) Species richness |
| 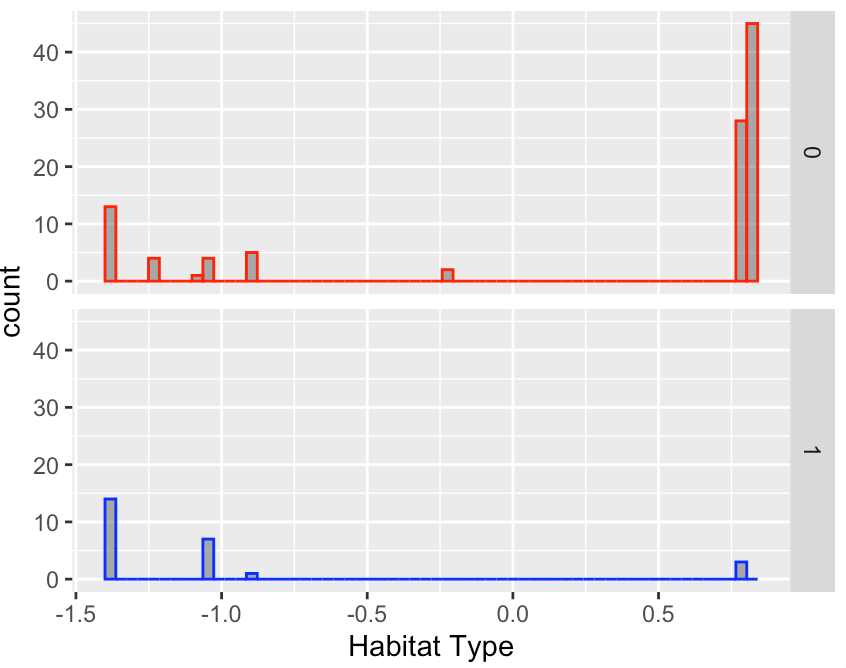 | 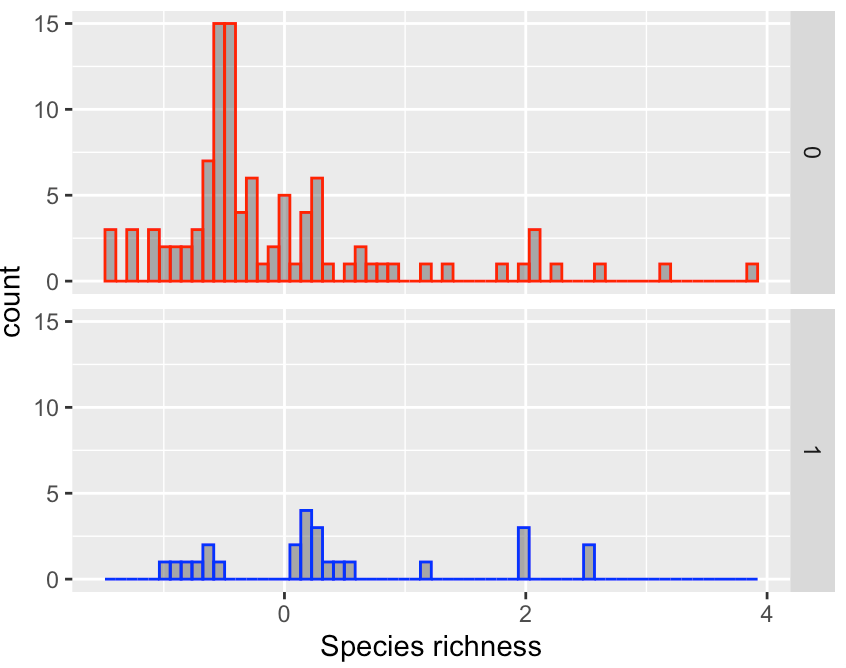 |
| e) Human population density (people/km^2^) |  |
| 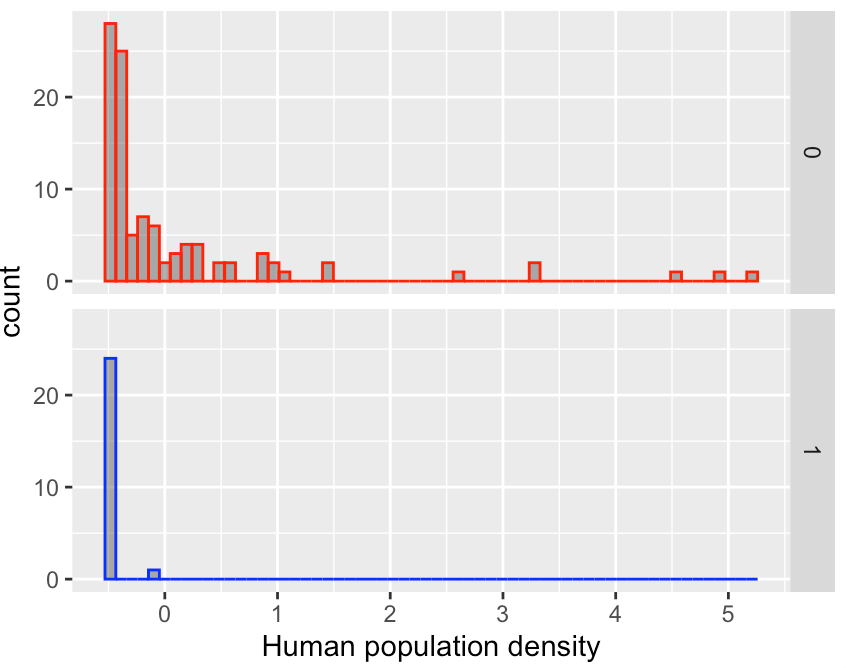 |  |

**S1 Table. Univariable analysis of putative risk factors for *T. gondii* oocyst shedding in free-ranging domestic and wild felids**

| Confirmed *T. gondii* studies (n=50) | | | | |
| --- | --- | --- | --- | --- |
| Variable Name | Effect Size Domestic (n=38) | p-value | Effect Size Wild (n=12) | p-value |
| Annual mean temperature | 0.30 (-0.07, 0.66) | 0.11 | -0.33 (-1.00, 0.35) | 0.34 |
| Mean diurnal range | **0.52 (0.21, 0.82)** | **0.00073** | -0.33 (-0.10, 1.26) | 0.09 |
| Isothermality | **0.30 (0.03, 0.57)** | **0.0015** | -0.28 (-1.17, 0.62) | 0.544 |
| Temperature seasonality | -0.25 (-0.55, 0.05) | 0.10 | **1.52 (0.71, 2.34)** | **0.00025** |
| Max warm month | 0.24 (-0.12, 0.60) | 0.19 | 0.09 (-0.67, 0.84) | 0.82 |
| Min cold month | 0.22 (-0.10, 0.55) | 0.17 | -0.74 (-1.50, 0.30) | 0.06 |
| Temperature annual range | -0.12 (-0.45, 0.20) | 0.47 | **1.25 (0.74, 1.76)** | **1.38e-06** |
| Mean wet quarter | 0.03 (-0.003, 0.06) | 0.078 | -0.06 (-0.14, 0.02) | 0.14 |
| Mean dry quarter | 0.27 (-0.07, 0.60) | 0.115 | **-1.06 (-1.69, -0.43)** | **0.00099** |
| Annual precipitation | -0.20 (-0.69, 0.29) | 0.42 | -0.08 (-0.53, 0.37) | 0.73 |
| Precipitation seasonality | **0.50 (0.14, 0.87)** | **0.007** | -0.33 (-1.09, 0.43) | 0.39 |
| Precipitation warm quarter | -0.29 (-0.62, 0.05) | 0.09 | 0.06 (-0.41, 0.53) | 0.80 |
| Precipitation cold quarter | -0.18 (-0.65, 0.30) | 0.46 | -0.25 (-0.81, 0.30) | 0.37 |
| Precipitation wet quarter | 0.0007 (-0.0008, 0.002) | 0.42 | -0.0005 (-0.003, 0.002) | 0.70 |
| Precipitation dry quarter | -0.47 (-0.90, -0.05) | 0.027 | -0.05 (-0.42, 0.33) | 0.81 |
| Precipitation wet month | 0.0015 (-0.003, 0.006) | 0.57 | -0.002 (-0.009, 0.005) | 0.64 |
| Precipitation dry month | -0.44 (-0.87, -0.004) | 0.048 | -0.05 (-0.42, 0.32) | 0.79 |
| Vapor pressure | 0.09 (-0.22, 0.40) | 0.57 | -0.05 (-1.15, 1.06) | 0.94 |
| Human footprint | 0.33 (-0.14, 0.80) | 0.17 | 0.26 (-0.78, 1.30) | 0.63 |
| Habitat type | 0.02 (-0.14, 0.18) | 0.77 | 0.06 (-0.29, 0.41) | 0.74 |
| Human population density | **0.40 (0.17, 0.63)** | **0.00058** | **4.14 (-0.10, 8.39)** | **0.056** |
| Species richness | 0.20 (-0.09, 0.49) | 0.18 | 0.48 (-0.78, 1.75) | 0.45 |
| Latitude | -0.003 (-0.015, 0.009) | 0.60 | -0.02 (-0.04, 0.008) | 0.20 |

Coefficients are shown as betas with a 95% confidence interval and p-value. Variables in bold were the most significant as determined by p-value, and were evaluated in subsequent multivariable models.

**S2 Table. Pearson correlation coefficients of significant univariable variables prior to multivariable model building.**

a. Domestic

|  | Isothermality | Mean diurnal range | Precipitation seasonality | Human population density |
| --- | --- | --- | --- | --- |
| Isothermality |  |  |  |  |
| Mean diurnal range | 0.12 |  |  |  |
| Precipitation seasonality | 0.31 | 0.67 |  |  |
| Human population density | -0.0096 | 0.12 | 0.21 |  |

b. Wild

|  | Temperature seasonality | Temperature annual range | Mean temp. dry quarter | Human population density |
| --- | --- | --- | --- | --- |
| Temperature seasonality |  |  |  |  |
| Temperature annual range | 0.94 |  |  |  |
| Mean temp. dry quarter | -0.56 | -0.47 |  |  |
| Human population density | -0.015 | 0.004 | 0.20 |  |

2(a) reflects coefficients between variables assessed for free-ranging domestic cats, and 2(b) reflects coefficients between variables assessed for wild felids. All temperature variables for wild felids (temperature seasonality, temperature annual range, mean temperature in driest quarter) were tested in separate models due to the high level of correlation between candidate variables.

**S3 Table. Model selection results based on Akaike information criterion (AIC) for testing hypotheses for climate (temperature and precipitation) and anthropogenic (human population density) factors associated with *Toxoplasma gondii* oocyst shedding prevalence in free-ranging domestic cats.**

|  |  | AIC | ΔAIC |
| --- | --- | --- | --- |
| (1\|study) + human population density + meandiurnalrange | Final model | -80.4 | 0 |
| (1\|study) + human population density + meandiurnalrange + precipitation seasonality |  | -79.4 | 1 |
| (1\|study) + human population density + isothermality + precipitation seasonality |  | -77.9 | 2.6 |
| (1\|study) + human population density + precipitation seasonality |  | -77.9 | 2.6 |
| (1\|study) + meandiurnalrange |  | -76.6 | 3.9 |
| (1\|study) + meandiurnalrange + precipitation seasonality |  | -75.7 | 4.8 |
| (1\|study) + precipitation seasonality + isothermality |  | -75.0 | 5.5 |
| (1\|study) + human population density + isothermality |  | -75.0 | 5.5 |
| (1\|study) + precipitation seasonality |  | -74.3 | 6.2 |
| (1\|study) + human population density |  | -73.5 | 6.9 |
| (1\|study) + isothermality |  | -72.0 | 8.5 |
| (1\|study) |  | -69.7 | 10.8 |

ΔAIC = change in AIC relative to the final model

**S4 Table. Wild felid model selection results based on Akaike information criterion (AIC) for testing hypotheses for climate (temperature and precipitation) and anthropogenic (human population density) factors associated with *Toxoplasma gondii* oocyst shedding prevalence in free-ranging wild felids.**

|  |  | AIC | ΔAIC |
| --- | --- | --- | --- |
| (1\|study) + human population density + meandryquarter | Final model | -1.1 | 0 |
| (1\|study) + human population density + temp seasonality |  | -1.1 | 0 |
| (1\|study) + human population density + temp annual range |  | 1.7 | 2.9 |
| (1\|study) + temp annual range |  | 5 | 6.2 |
| (1\|study) + temp seasonality |  | 7.8 | 9 |
| (1\|study) + meandryquarter |  | 8.9 | 10.1 |
| (1\|study) + human population density |  | 12.4 | 13.5 |
| (1\|study) |  | 12.8 | 14 |

ΔAIC = change in AIC relative to the final model
