# Supplementary material for: More people, more cats, more parasites: Human population density and temperature variation predict prevalence of Toxoplasma gondii oocyst shedding in free-ranging domestic and wild felids
Source: PLoS One. 2023 Jun 21;18(6):e0286808. doi: 10.1371/journal.pone.0286808 (PMC10284397; doi:10.1371/journal.pone.0286808)
Supplement: S1 Appendix — (DOCX) [file pone.0286808.s002.docx]

Supporting Information Appendix S1. List of published references used in global analysis of oocyst shedding in free-ranging wild and domestic felids.

1. Ahn, K.-S. *et al.* Excretion of *Toxoplasma gondii* oocysts from Feral Cats in Korea. *Korean J. Parasitol.* **57**, 665–670 (2019).

2. Akuzawa, M., Mochizuki, M. & Yasuda, N. Hematological and parasitological study of the Iriomote cat (*Prionailurus iriomotensis*). *Can. J. Zool.* **65**, 946–949 (1987).

3. Al-Kappany, Y. M. *et al.* High Prevalence of Toxoplasmosis in Cats from Egypt: Isolation of Viable *Toxoplasma gondii*, Tissue Distribution, and Isolate Designation. *J. Parasitol.* **96**, 1115–1118 (2010).

4. Aramini, J. J. *et al.* Potential contamination of drinking water with *Toxoplasma gondii* oocysts. *Epidemiol. Infect.* **122**, 305–315 (1999).

5. Aramini, J. J., Stephen, C. & Dubey, J. P. *Toxoplasma gondii* in Vancouver Island Cougars (*Felis concolor vancouverensis*): Serology and Oocyst Shedding. *J. Parasitol.* **84**, 438–440 (1998).

6. Asgari, Q. *et al.* Molecular and Serological Detection of *Toxoplasma gondii* in Stray Cats in Shiraz, South-central, Iran. *Iran. J. Parasitol.* **13**, 430–439 (2018).

7. Berger-Schoch, A. E. *et al.* Prevalence and genotypes of *Toxoplasma gondii* in feline faeces (oocysts) and meat from sheep, cattle and pigs in Switzerland. *Vet. Parasitol.* **177**, 290–297 (2011).

8. Brown, M., Lappin, M. R., Brown, J. L., Munkhtsog, B. & Swanson, W. F. Exploring the ecologic basis for extreme susceptibility of Pallas’ cats (*Otocolobus manul*) to fatal toxoplasmosis. *J. Wildl. Dis.* **41**, 691–700 (2005).

9. Carver, S. *et al.* Zoonotic Parasites of Bobcats around Human Landscapes. *J. Clin. Microbiol.* **50**, 3080–3083 (2012).

10. Chi, X., Fang, K., Koster, L., Christie, J. & Yao, C. Prevalence of Feline Immunodeficiency Virus and *Toxoplasma gondii* in Feral Cats on St. Kitts, West Indies. *Vet. Sci.* **8**, (2021).

11. Dubey, J. P., Christie, E., & Pappas, P. W. Characterization of *Toxoplasma gondii* from the feces of naturally infected cats. *J. Infect. Dis.* **136**, 432–435 (1977).

12. de Aluja, A. S. & Aguilar, P. Estudio sobre la frecuencia del ooquiste de *Toxoplasma gondii* en el gato domestico del distrito federal. *Gac. Médica Mex.* **113**, 455–459 (1977).

13. Dubey, J. P. *et al.* Genetic and biologic characterization of *Toxoplasma gondii* isolates of cats from China. *Vet. Parasitol.* **145**, 352–356 (2007).

14. Dubey, J. P. *et al.* Prevalence of *Toxoplasma gondii* in cats from Colombia, South America and genetic characterization of *T. gondii* isolates. *Vet. Parasitol.* **141**, 42–47 (2006).

15. Dubey, J. P. *et al.* Isolation of viable *Toxoplasma gondii* from tissues and feces of cats from Addis Ababa, Ethiopia. *J. Parasitol.* **99**, 56–58 (2013).

16. Dubey, J. P. *et al.* Genetic diversity of *Toxoplasma gondii* isolates from Ethiopian feral cats. *Vet. Parasitol.* **196**, 206–208 (2013).

17. Dubey, J. P. *et al.* Isolation and characterization of viable *Toxoplasma gondii* isolates revealed possible high frequency of mixed infection in feral cats (*Felis domesticus*) from St Kitts, West Indies. *Parasitology* **136**, 589–594 (2009).

18. Dubey, J. P. *et al.* Sources and reservoirs of *Toxoplasma gondii* infection on 47 swine farms in Illinois. *J. Parasitol.* **81**, 723–729 (1995).

19. Dubey, J. P. *et al.* Mouse-virulent *Toxoplasma gondii* isolated from feral cats on Mona Island, Puerto Rico. *J. Parasitol.* **93**, 1365–1369 (2007).

20. Frenkel, J. K. *et al.* Transmission of *Toxoplasma gondii* in Panama City, Panama: a five-year prospective cohort study of children, cats, rodents, birds, and soil. *Am. J. Trop. Med. Hyg.* **53**, 458–468 (1995).

21. Hata, H. *et al.* Prevalence of *Toxoplasma gondii* and other intestinal parasites in cats in Chiba Prefecture, Japan. *Jpn. J. Trop. Med. Hyg.* **28**, 365–368 (2000).

22. Lilly, E. L. & Wortham, C. D. High prevalence of *Toxoplasma gondii* oocyst shedding in stray and pet cats (*Felis catus*) in Virginia, United States. *Parasit. Vectors* **6**, 266 (2013).

23. Marchiondo, A. A., Duszynski, D. W. & Maupin, G. O. Prevalence of antibodies to *Toxoplasma gondii* in wild and domestic animals of New Mexico, Arizona, and Colorado. *J. Wildl. Dis.* **12**, 226–232 (1976).

24. McKenna, P. B. & Charleston, W. A. Coccidia (*Protozoa:Sporozoasida*) of cats and dogs. I. Identity and prevalence in cats. *N. Z. Vet. J.* **28**, 86–88 (1980).

25. Jackson, M. H., Hutchison, W. M. & Siim, J. C. Prevalence of *Toxoplasma gondii* in meat animals, cats and dogs in central Scotland. *Br. Vet. J.* **143**, 159–165 (1987).

26. Oikawa, H. *et al.* Survey on *Toxoplasma* infection in stray cats in western area of Japan during a two-year period. *Jpn. J. Parasitol.* **39**, 462–467 (1990).

27. Pampiglione, S., Poglayen, G., Arnone, B. & De Lalia, F. *Toxoplasma gondii* oocysts in the faeces of naturally infected cat. *Br. Med. J.* **2**, 306 (1973).

28. Pena, H. F. J., Soares, R. M., Amaku, M., Dubey, J. P. & Gennari, S. M. *Toxoplasma gondii* infection in cats from São Paulo state, Brazil: Seroprevalence, oocyst shedding, isolation in mice, and biologic and molecular characterization. *Res. Vet. Sci.* **81**, 58–67 (2006).

29. Pizzi, H. L., Rico, C. M. & Pessat, O. A. N. Hallazgo del ciclo ontogenico selvatico del *Toxoplasma gondii* en felidos salvajes (*Oncifelis geofroyi, Felis colocolo* y *Felis eirá*) de la Provincia de Cordoba. *Rev. Mil. Vet.* **25**, 293–300 (1978).

30. Pop, A., Cerbu, A., Pop, A. & Andreescu, N. The seasonal prevalence of the *Toxoplasma gondii* infections in stray cats from urban area studied by parasitological methods. *Arch. Roum. Pathol. Exp. Microbiol.* **45**, 57–63 (1986).

31. Razmi, Gh. R. Prevalence of Feline Coccidia in Khorasan Province of Iran. *J. Appl. Anim. Res.* **17**, 301–303 (2000).

32. Rifaat, M. A. *et al.* *Toxoplasma* infection of stray cats in Egypt. *J. Trop. Med. Hyg.* **79**, 67–70 (1976).

33. Rothe, J., McDonald, P. J. & Johnson, A. M. Detection of *Toxoplasma* Cysts and Oocysts in an Urban Environment in a Developed Country. *Pathology (Phila.)* **17**, 497–499 (1985).

34. Roudná, V. [Examination of cats for the elimination of *Toxoplasma gondii* oocysts]. *Vet. Med. (Praha)* **24**, 59–64 (1979).

35. Ruiz, A. & Frenkel, J. K. *Toxoplasma gondii* in Costa Rican Cats*. *Am. J. Trop. Med. Hyg.* **29**, 1150–1160 (1980).

36. Salant, H., Markovics, A., Spira, D. T. & Hamburger, J. The development of a molecular approach for coprodiagnosis of *Toxoplasma gondii*. *Vet. Parasitol.* **146**, 214–220 (2007).

37. Shastri, U. V. & Ratnaparkhi, M. R. *Toxoplasma* and other intestinal coccidia in cats in Maharashtra (Parbhani). *Indian Vet. J.* **69**, 14–16 (1992).

38. Sroka, J. *et al.* Prevalence of *Toxoplasma gondii* infection in cats in southwestern Poland. *Ann. Agric. Environ. Med.* **25**, 576–580 (2018).

39. Tavalla, M., Asgarian, F. & Kazemi, F. Prevalence and genetic diversity of *Toxoplasma gondii* oocysts in cats of southwest of Iran. *Infect. Dis. Health* **22**, 203–209 (2017).

40. VanWormer, E. *et al.* *Toxoplasma gondii*, Source to Sea: Higher Contribution of Domestic Felids to Terrestrial Parasite Loading Despite Lower Infection Prevalence. *EcoHealth* **10**, 277–289 (2013).

41. Venturini, L., Venturini, M. C. & Omata, Y. Diagnostico de toxoplasmosis durante el periodo patente en un gato domestico. *Vet. Argent.* **9**, 528–531 (1992).

42. Verma, S. K. *et al.* Seroprevalence, isolation and co-infection of multiple *Toxoplasma gondii* strains in individual bobcats (*Lynx rufus*) from Mississippi, USA. *Int. J. Parasitol.* **47**, 297–303 (2017).

43. Verma, S. K. *et al.* Antibody Detection and Molecular Characterization of *Toxoplasma gondii* from Bobcats (*Lynx rufus*), Domestic Cats (*Felis catus*), and Wildlife from Minnesota, USA. *J. Eukaryot. Microbiol.* **63**, 567–571 (2016).

44. Wallace, G. D. The role of the cat in the natural history of *Toxoplasma gondii*. *Am. J. Trop. Med. Hyg.* **22**, 313–322 (1973).

45. Werner, J. K. & Walton, B. C. Prevalence of naturally occurring *Toxoplasma gondii* infections in cats from U.S. military installations in Japan. *J. Parasitol.* **58**, 1148–1150 (1972).

46. Yang, Y. *et al.* Isolation and genetic characterization of viable *Toxoplasma gondii* from tissues and feces of cats from the central region of China. *Vet. Parasitol.* **211**, 283–288 (2015).

47. Zástĕra, M., Pokorný, J. & Sedlácková, H. [Isolation of *Toxoplasma gondii* strains from faeces of stray cats in Czechoslovakia (author’s transl)]. *Bratisl. Lek. Listy* **68**, 57–67 (1977).
